# Secondhand smoke exposure and current tobacco and/or nicotine use among adolescents in Kenya and Nigeria: a survey-weighted inverse probability weighting analysis

**DOI:** 10.1136/bmjph-2025-004773

**Published:** 2026-10-13

**Authors:** Grace Kyule, Shukri F. Mohamed, Lyagamula Kisia, Olatunbosun Abolarin, Uche Okezie, Nelson Mbaya, Akinsewa Akiode, Thompson Ademola, James Kavai, Franklin Koech, Bosco Okumu, Damazo T. Kadengye, Samuel Iddi

**Affiliations:** 1 African Population and Health Research Center, Nairobi, Kenya; 2 APIN Public Health Initiatives, Abuja, Nigeria; 3 Research and Communications Services (RCS), Abuja, Nigeria; 4 Department of Statistics and Actuarial Science, University of Ghana, Accra, Ghana

**Keywords:** Adolescent, Public Health, Causality, Cross-Sectional Studies

## Abstract

**Introduction:**

Secondhand smoke (SHS) exposure is associated with adolescent tobacco and nicotine use but nationally representative evidence from sub-Saharan Africa is limited. We examined this association among adolescents in Kenya and Nigeria using survey-weighted inverse probability weighting.

**Methods:**

We analysed cross-sectional data from the 2024 Data on Youth Tobacco in Africa household survey of adolescents aged 10–17 years in Kenya (n=6435) and Nigeria (n=7502). SHS exposure was self-reported. Current use of any tobacco and/or nicotine product was self-reported past-30-day use of cigarettes, roll-your-own tobacco, shisha, smokeless tobacco, heated tobacco products, e-cigarettes or nicotine pouches. We estimated the average treatment effect (ATE) and average treatment effect on the treated (ATT), adjusting for age, sex, residence, school attendance, adolescent employment, household head education, household size, wealth and country in pooled analyses. We assessed effect modification by country and conducted regression, location-specific and E-value sensitivity analyses.

**Results:**

Overall, 38.2% reported SHS exposure and 3.1% reported current use; 72.1% of current users were boys. Current use was more common among SHS-exposed than unexposed adolescents (4.4% vs 2.4%). In pooled analyses, SHS exposure was associated with a 2.65 percentage-point higher probability of current use (ATE: +2.65 pp; 95% CI 1.46 to 3.83); the ATT was 2.42 percentage points (95% CI 1.35 to 3.49). The ATE was larger in Nigeria (+3.94 pp; 95% CI 1.95 to 5.93) than Kenya (+1.32 pp; 95% CI 0.19 to 2.45), with evidence of effect modification (interaction p=0.019). Sensitivity analyses were consistent, although unmeasured confounding remains possible.

**Conclusions:**

SHS exposure was associated with a higher probability of adolescent tobacco and/or nicotine use. Because exposure and outcome were measured cross-sectionally, temporality and causality cannot be established. Strengthening smoke-free policies may help reduce adolescent SHS exposure and tobacco and/or nicotine use.

**WHAT IS ALREADY KNOWN ON THIS TOPIC**

- Secondhand smoke (SHS) exposure is a known risk factor for adolescent tobacco initiation. Although many sub-Saharan African countries have adopted WHO Framework Convention on Tobacco Control (FCTC) measures, enforcement of comprehensive smoke-free environments remains uneven. Evidence on the relationship between SHS exposure and adolescents’ tobacco and/or nicotine use in African settings is limited, particularly from nationally representative data.

**WHAT THIS STUDY ADDS**

- Using nationally representative data from Kenya and Nigeria, this study provides survey-weighted inverse probability weighting estimates of the association between SHS exposure and current tobacco and/or nicotine use among adolescents. SHS exposure was associated with a higher probability of current use, with a larger association observed in Nigeria than Kenya.

**HOW THIS STUDY MIGHT AFFECT RESEARCH, PRACTICE OR POLICY**

- The observed association between SHS exposure and adolescent tobacco and/or nicotine use underscores the need to strengthen enforcement of WHO FCTC Article 8 smoke-free policies. These findings support targeted preventive interventions aimed at reducing adolescent exposure and shaping healthier social norms around tobacco and/or nicotine use.

## Introduction

 Tobacco use remains a major cause of preventable morbidity and mortality, with secondhand smoke (SHS) exposure contributing to this burden. The WHO estimates that SHS contributes to approximately 1.6 million annual deaths among people who do not smoke.^1,2^ Evidence from recent studies also suggests that SHS is associated with tobacco use susceptibility, initiation and current use, particularly among adolescents.^3,4^ Adolescents face both direct health risks of SHS and an increased susceptibility to tobacco and nicotine initiation.^5,6^

Adolescents have limited control over their environments and are often exposed to SHS involuntarily. Globally, more than 40% of young people encounter SHS in public spaces, while about 30% are exposed at home.^7–9^ In sub-Saharan Africa (SSA), and especially in Kenya and Nigeria, SHS exposure among adolescents remains widespread.^10,11^ National data show that 50.7% of adolescents in Kenya and 42.9% in Nigeria are exposed in public places, while 27.4% and 24.1%, respectively, report exposure within their homes.^12,13^ This widespread exposure not only puts adolescents at immediate health risk, but may also make tobacco and nicotine use seem socially acceptable or less harmful, which can increase the likelihood of tobacco use. This pathway is consistent with social learning theory,^14^ which suggests that adolescents may learn and adopt behaviours by observing and modelling behaviours within their social environment. In the context of SHS, repeated observation of smoking by adults, family members or peers may normalise tobacco and nicotine use, reduce perceived harm, increase curiosity or susceptibility, and create opportunities for access to tobacco or nicotine products.

The relationship between adolescent SHS exposure and tobacco use is shaped by several factors that may confound or mediate the association. Studies have shown that age, sex, household socio-economic status, school attendance, household composition (including the presence of extended family members or non-relatives) and parental education influence the likelihood of SHS exposure and the risk of initiating tobacco use.^5,15–18^ Adolescents in lower-income households or those with less-educated caregivers are more likely to encounter SHS and to use tobacco.^19,20^ Similarly, the smoking behaviour of parents, siblings or peers has been shown to play a crucial role in shaping adolescents’ perceptions of tobacco.^18,21,22^

Despite high levels of SHS exposure in Kenya and Nigeria, there is limited nationally representative evidence on the relationship between SHS exposure and adolescent tobacco and/or nicotine use.^10^ Most studies in the region rely on school-based surveys that exclude out-of-school adolescents and report associative findings rather than estimates based on causal-inference methods.^9,10^ Few have applied methods such as inverse probability weighting (IPW) to account for measured confounding while retaining the nationally representative survey design features. In addition, to our knowledge no nationally representative study has assessed whether the association between SHS exposure and adolescent tobacco and/or nicotine use differs across African settings, despite variations in tobacco control policies, enforcement capacity and sociocultural contexts that may influence both exposure patterns and behaviour. Kenya and Nigeria also differ in the maturity and enforcement of smoke-free protections, which may influence where adolescents are exposed to SHS and how such exposure relates to tobacco and/or nicotine use.

This study addresses these gaps by examining the association between SHS exposure and adolescent tobacco and/or nicotine use in Kenya and Nigeria using survey-weighted IPW to better account for measured confounding and estimate adjusted risk differences within a causal inference framework. We also assessed whether the magnitude of this association varies between the two countries. Guided by a directed acyclic graph (DAG)-based conceptual framework, we hypothesised that adolescents exposed to SHS would have a higher probability of current tobacco and/or nicotine use than unexposed adolescents after adjustment for measured demographic, socio-economic and household factors, and that the magnitude of this association may differ between Kenya and Nigeria because of differences in exposure context, enforcement and social norms. Because exposure and outcome were measured in the same cross-sectional survey, these estimates are interpreted cautiously and should not be considered definitive evidence of causality because temporality cannot be established. Their interpretation depends on several causal-inference assumptions, including conditional exchangeability, positivity, consistency and correct model specification. The findings provide policy-relevant evidence to inform smoke-free protections and support implementation of Article 8 of the WHO Framework Convention on Tobacco Control (FCTC), which mandates comprehensive measures to protect populations from tobacco smoke.

## Methods

### Study design and sample

This study used data from the 2024 Data on Youth Tobacco in Africa (DaYTA) survey, a nationally representative cross-sectional household survey of adolescents aged 10–17 years in Kenya, Nigeria and the Democratic Republic of the Congo (DRC). The survey employed a multistage, stratified cluster sampling design, selecting one adolescent per household. Survey weights were applied to account for the complex sampling design, adjust for unequal selection probabilities and approximate national representativeness. Full details on the study design, recruitment and sample size determination are available in a published protocol.^23^ The deidentified DaYTA dataset used for this analysis is available through the African Population and Health Research Center (APHRC) Microdata Portal.^24^

The present secondary analysis was restricted to Kenya and Nigeria. The APHRC-led study team was responsible for managing and analysing data from these two countries, while the DRC collaborating team retained stewardship of the DRC dataset. Furthermore, when this analysis was initiated, the DRC dataset was not yet available to our analytical team in a final analysis-ready form. Data collection in Kenya and Nigeria was conducted between April and June 2024. The survey response rate was 96% in Kenya and 94% in Nigeria.

The final descriptive sample comprised 13 937 adolescents, including 6435 from Kenya and 7502 from Nigeria. The primary IPW analyses followed a complete-case approach for the exposure, outcome, covariates, sampling weights and primary sampling units (PSUs) resulting in an analytical sample of 12 216 adolescents. SHS exposure and current tobacco and/or nicotine use data were available for all adolescents.

### Variables and measurements

The primary exposure was SHS exposure, defined as a binary variable indicating whether the adolescent was exposed to tobacco smoke from others in measured settings. SHS exposure was assessed using self-reported setting-specific questions on exposure to people smoking in the home and in public or institutional settings, including public transportation and school, university or health-facility buildings. Exposure inside the home was assessed over the past 7 days, whereas exposure inside public transportation and school, university or health-facility buildings was assessed over the past 30 days. Because the analysis focused on the occurrence of any exposure rather than exposure frequency, each setting-specific item was dichotomised within its original recall period and combined without rescaling to a common time period. The resulting composite represented any recent SHS exposure across the measured settings, with recall periods ranging from 7 to 30 days. Adolescents were classified as ‘exposed to SHS’ if they reported any exposure to tobacco smoke in at least one measured setting and ‘not exposed’ if they reported no exposure in all settings.

The outcome of interest was current use of any tobacco and/or nicotine product. This was defined as a binary indicator of whether the adolescent had used any tobacco and/or nicotine product in the past 30 days. The products covered both smoked and smokeless tobacco including manufactured cigarettes, hand-rolled cigarettes, shisha (waterpipe hookah), smokeless tobacco (eg, chew, snuff), other smoked tobacco (cigars), heated tobacco products, electronic cigarettes (e-cigarettes/vape) and nicotine pouches. An adolescent who reported use of one or more of these products in the past 30 days was classified as a current tobacco and/or nicotine user (coded as 1) and those who indicated no use of any listed product were classified as non-users (coded as 0). This outcome reflects current use rather than incident initiation. Therefore, adolescents who first used any tobacco and/or nicotine product within the past 30 days would be included as current users if they reported at least 1 day of use in the past 30 days; however, the primary outcome does not separately distinguish new initiators from continuing users.

All variables were collected using a harmonised DaYTA questionnaire administered uniformly in Kenya and Nigeria, ensuring comparability across settings. Both SHS exposure and tobacco and/or nicotine use were self-reported.

### Causal framework and assumptions

We developed a DAG to guide covariate selection and clarify the assumed relationship between SHS exposure and current tobacco and/or nicotine use (online supplemental figure 1). The DAG conceptualised SHS exposure as a social and environmental exposure that may be associated with current tobacco and/or nicotine use through pathways such as social normalisation, reduced perceived harm, susceptibility and access to tobacco or nicotine products. The DAG identified potential confounding variables (common causes of SHS exposure and tobacco/nicotine use) that were measured in the survey and adjusted for in the analysis. These included demographic characteristics, socio-economic position, household composition, school status, employment and in the pooled analysis, country.

Peer smoking and family or parental tobacco use were also considered important potential confounders because they may influence both adolescents’ exposure to SHS and their likelihood of tobacco or nicotine use. However, these variables were not included in the primary propensity score model because they were not available for the full analytical sample. Including them would have restricted the analysis to a selected subgroup and changed the target population. We therefore treated peer smoking and family or parental tobacco use as potential sources of residual unmeasured confounding and assessed their possible influence through E-value sensitivity analysis.

The IPW estimates were interpreted under the assumptions of conditional exchangeability, positivity, consistency and correct model specification. Conditional exchangeability assumes that, after adjustment for measured covariates, adolescents exposed and unexposed to SHS are comparable with respect to factors influencing current tobacco/nicotine use. Positivity assumes that adolescents in all covariate strata had a non-zero probability of being exposed and unexposed to SHS. Consistency assumes that the binary SHS exposure definition corresponds to a meaningful contrast between any self-reported exposure and no self-reported exposure across the measured settings. Correct model specification assumes that the propensity score model adequately captured the relationship between covariates and SHS exposure. Because exposure and outcome were measured cross-sectionally, temporality could not be confirmed and reverse causation remains possible.

### Confounders

Potential confounders were selected based on a priori knowledge and the DAG-based causal framework of factors associated with both SHS exposure and tobacco and/or nicotine use in adolescents. To make more plausible the conditional exchangeability assumption, a prerequisite for causal inference^25^ the following covariates were included, age group (10–13 or 14–17 years), sex (boy/girl), residence (urban or rural), school attendance status (in-school/out-of-school), adolescent employment status (employed/not employed), household head’s education (primary or below vs secondary or higher), household size (continuous), a composite asset-based wealth index stratified into quintiles (constructed via principal component analysis) and country (Kenya/Nigeria) to adjust for unmeasured country-level differences in tobacco control context, culture and baseline prevalence in the pooled analysis. These variables were included because they may influence both adolescents’ likelihood of being exposed to SHS and their likelihood of using tobacco and/or nicotine products. Age and sex were included because tobacco and/or nicotine use and social exposure patterns vary by developmental stage and gender. Residence was included because urban and rural adolescents may differ in public-space exposure, product availability and enforcement of smoke-free policies. School attendance and employment status were included because they capture adolescents’ social environments, supervision, mobility and access to tobacco/nicotine products. Household head’s education, household size and wealth were included as markers of household socio-economic position and living conditions, which may influence both exposure to people who smoke and tobacco/nicotine use. Country was included in pooled models to account for broader differences in tobacco-control context, enforcement, social norms and baseline prevalence between Kenya and Nigeria.

Most covariates were demographic, household or socio-economic characteristics that were considered fixed or relatively stable at the time of the survey. School attendance and employment status were measured at the time of the survey and were treated as contextual confounders, although the cross-sectional design means their temporal ordering relative to SHS exposure and current tobacco/nicotine use cannot be confirmed with certainty.

### Participants and public involvement

Members of the public, including youth and adolescent advocates, were involved in the design, conduct and dissemination of the primary DaYTA study from which this analysis is derived. During the development of the DaYTA study, youth advocates, together with representatives from ministries of health and education, national statistical offices and civil society organisations in Kenya and Nigeria, contributed through stakeholder consultations that informed study priorities, questionnaire development and implementation procedures. Data collection was undertaken by trained fieldworkers recruited from the study regions to enhance contextual appropriateness and acceptability. Following completion of data collection, study findings were disseminated through national and regional stakeholder forums that included policymakers, public health practitioners, youth advocates and adolescent representatives. This manuscript reports a secondary analysis of the DaYTA survey data; patients were not involved, and members of the public were not involved in the statistical analysis or writing of this manuscript.

### Analytical methods

#### Survey-weighted inverse probability weighting analysis

To examine the association between SHS exposure and adolescent tobacco and/or nicotine use, and to generate risk-difference estimates with a causal interpretation under specified assumptions, a survey-weighted IPW approach was implemented within the potential outcomes’ framework. This method accounts for observed confounding while respecting the complex sampling design of the source surveys. First, propensity scores (the conditional probability of SHS exposure given covariates) were estimated using logistic regression. Each model (separately for the pooled sample and each country) included all a priori covariates namely age group, sex, residence, school status, employment, household head education, household size, wealth index and country (for pooled analyses) to account for observed heterogeneity. Survey sampling weights were incorporated when estimating propensity scores to preserve population-representativeness. The final IPW analysis weight was calculated by multiplying the survey sampling weight by the estimated treatment weight. This combined weight allowed the analysis to adjust for both the complex survey design and measured differences in the probability of SHS exposure. Variance estimation accounted for the multistage design using survey strata and PSUs, with Taylor-linearised robust SEs. We used a survey design-based rather than a mixed-effects approach because the PSUs were sampling-design units and the target estimands were marginal, population-average risk differences.

For estimation of the average treatment effect (ATE; the population-average risk differences for SHS exposure under the stated assumptions) and the average treatment effect on the treated (ATT; the risk differences among adolescents exposed to SHS under the stated assumptions), we used IPWs based on the propensity score. The IPW weights were defined as:


$$w_{ATE}=\frac{Z}{ê\left(X\right)}\;+\;\frac{1-Z}{1-ê\left(X\right)},\;w_{ATT}=Z\;+\;\left(1-Z\right)\frac{ê\left(X\right)}{1-ê\left(X\right)}$$

where *Z* is a binary treatment indicator for SHS exposure (1=exposed, 0=not exposed), and *ê(X*) is the estimated propensity score (the predicted probability of being exposed to SHS conditional on observed covariates *X*). The corresponding risk differences were then estimated as:


$$\begin{array}{l} ATE=\frac{\sum_{i}w_{ATE,i}Z_{i}Y_{i}}{\sum_{i}w_{ATE,i}Z_{i}}-\frac{\sum_{i}w_{ATE,i}(1-Z_{i})Y_{i}}{\sum_{i}w_{ATE,i}(1-Z_{i})},\\ \;and\;ATT=\frac{\sum_{i}Z_{i}Y_{i}}{\sum_{i}Z_{i}}-\frac{\sum_{i}w_{ATT,i}(1-Z_{i})Y_{i}}{\sum_{i}w_{ATT,i}(1-Z_{i})} \end{array}$$

where, $Y_{i}$ is the outcome variable (current use of any tobacco and/or nicotine), $Z_{i}$ is exposure indicator (1=exposed, 0=not exposed) and $w_{ATE,i}$*,* $w_{ATT,i}$ are the weights for ATE and ATT, respectively. The summations ($\sum_{i}$) are taken over all adolescents *i* in the sample.

#### Covariate balance, overlap and weight diagnostics

Covariate balance was assessed preweighting and postweighting using standardised mean differences (SMDs) and variance ratios. Balance was achieved when |SMD| <0.10 and variance ratios fell within (0.80–1.25), consistent with established guidelines.^25,26^ We generated Love plots to visually compare covariate balance before and after weighting. We examined the distribution of estimated propensity scores by exposure group to assess common support and overlap between exposed and unexposed adolescents. We also inspected the distribution of IPW weights, including minimum, maximum, mean, SD and selected percentiles, to identify extreme weights. No weight truncation was applied because the treatment-weight diagnostics did not indicate extreme IPW weights.

#### Primary and supplementary analyses

For the primary analysis, ATE and ATT risk differences were estimated for the pooled sample and separately for Kenya and Nigeria. CIs were derived using Taylor-linearised robust variance estimators that accounted for clustering at the PSU level and incorporated survey design features. Statistical significance was evaluated at α=0.05 (two-tailed).

Descriptive analyses were survey-weighted and presented as weighted percentages with unweighted frequencies. Continuous variables were summarised using mean and SD or median and IQR, depending on distribution. Household size was summarised using median and IQR. P values less than 0.001 were reported as <0.001.

To assess effect modification by country, we fitted an IPW-weighted risk-difference model with an interaction term between SHS exposure and country. We estimated country-specific risk differences and the Nigeria–Kenya difference in risk differences under both the ATE and ATT specifications. The interaction p value was used to assess whether the association between SHS exposure and current tobacco and/or nicotine use differed between Kenya and Nigeria on the additive scale.

To assess the robustness of the primary findings, we fitted survey-weighted multivariable logistic regression models to estimate adjusted ORs (AORs) for the association between SHS exposure and current tobacco and/or nicotine use, incorporating sampling weights and adjusting for the same covariates as in the IPW models. Standard errors accounted for PSU-level clustering. We also derived marginal probabilities of tobacco and/or nicotine use by exposure status from these models and calculated adjusted risk differences using predictive margins, enabling comparison with the IPW-derived estimates.

To explore whether the binary SHS exposure definition masked differences by exposure context, we conducted an additional location-specific analysis using self-reported exposure at home and in public places. We created a four-category exposure–location variable: no SHS exposure, home-only exposure, public-place-only exposure and exposure both at home and in public places. We then fitted survey-weighted logistic regression models adjusted for the same covariates as the primary analysis and estimated adjusted predicted probabilities and risk differences across these exposure-location categories. This analysis was treated as exploratory because exposure settings may differ in duration, intensity and social meaning, and because the original cross-sectional survey was not designed to establish temporality across exposure contexts.

To assess the potential impact of unmeasured confounding by peer smoking and family or parental tobacco use, we calculated E-values using IPW-weighted risk ratios corresponding to the ATE estimates. E-values were calculated for the point estimate and for the confidence limit closest to the null. The E-value estimates the minimum strength of association that an unmeasured confounder would need to have with both SHS exposure and current tobacco and/or nicotine use, conditional on measured covariates, to fully explain away the observed association.

Analyses were conducted in Stata V.18.0 (StataCorp LLC, College Station, Texas, USA).

## Results

### Participant characteristics and SHS exposure

Table 1 shows proportions of SHS exposure by characteristics of adolescents. The full distribution of adolescents exposed and unexposed to SHS is provided in online supplemental table 1. Overall, prevalence of SHS exposure was 38.2% with differences between Kenya (48.7%) and Nigeria (29.2%). In the pooled sample, a higher proportion of adolescents exposed to SHS were aged 14–17 years, residing in rural areas, in school and engaged in employment. While exposure to SHS was observed across all wealth quintiles, the largest proportions were observed among those in the poor (40.8%) and middle (43.3%) quintiles. About 53% of current users were exposed to SHS compared with 37.7% of non-users in the pooled data.

**Table 1:** Survey-weighted prevalence of SHS exposure by participant characteristics, pooled and by country

	Pooled N (13 937)		Kenya N (6435)		Nigeria N (7502)	
	Exposed	P value	Exposed	P value	Exposed	P value
SHS exposure, n (%)	5331 (38.2)		3190 (48.7)		2141 (29.2)	
Age group, n (%)						
10–13 years	2888 (36.2)	0.006	1712 (47.8)	0.370	1176 (26.7)	0.008
14–17 years	2443 (40.6)		1478 (49.7)		965 (32.4)	
Sex, n (%)						
Girls	2561 (37.5)	0.245	1577 (46.0)	0.001	984 (29.6)	0.636
Boys	2770 (38.9)		1613 (51.5)		1157 (28.9)	
Residence, n (%)						
Rural	3420 (39.9)	0.010	2099 (49.0)	0.544	1321 (28.1)	0.312
Urban	1911 (35.5)		1091 (47.8)		820 (30.4)	
School status, n (%)						
Out of school	424 (27.0)	<0.001	143 (35.6)	<0.001	281 (23.6)	0.020
In school	4897 (40.1)		3045 (50.0)		1852 (30.5)	
Work status, n (%)						
Not employed	4827 (37.6)	0.005	2964 (47.8)	<0.001	1863 (28.4)	0.020
Employed	492 (44.7)		225 (61.5)		267 (36.2)	
Household head education, n (%)						
Primary and below	2353 (38.1)	0.536	1586 (47.1)	0.171	767 (27.4)	0.213
Secondary +	2312 (37.1)		1185 (50.0)		1127 (30.3)	
Wealth quintile, n (%)						
Poorest	949 (33.1)	<0.001	648 (43.2)	0.006	301 (24.5)	0.030
Poor	1155 (40.8)		794 (50.8)		361 (29.7)	
Middle	1169 (43.3)		715 (52.0)		454 (33.4)	
Rich	1121 (39.9)		641 (52.3)		480 (30.7)	
Richest	937 (35.2)		392 (47.7)		545 (30.0)	
Household size, median (IQR**)**	4 (3–5)		4 (3–5)		3 (3–5)	
Current tobacco and/or nicotine use, n (%)						
No	5076 (37.7)	<0.001	3111 (48.6)	0.463	1965 (28.3)	<0.001
Yes	255 (53.5)		79 (54.3)		176 (53.0)	

Values are unweighted frequencies (n) and survey-weighted percentages (%) unless otherwise stated. Percentages are row percentages showing the distribution of SHS exposure within each participant characteristic. P values are design-adjusted p values comparing adolescents exposed and not exposed to SHS within the pooled sample and within each country; p values <0.001 are reported as <0.001.

SHS, secondhand smoke.

Current tobacco and/or nicotine use was reported by 3.1% of adolescents in the pooled sample, 2.5% in Kenya and 3.7% in Nigeria. In the pooled analysis, current users were more likely to be aged 14–17 years (64.6%), boys (72.1%) and from the poorest wealth quintile (38.7%) compared with their respective counterparts. Country-specific patterns showed similar trends though with varying magnitudes. In Kenya, current users were more likely to be older adolescents (14–17 years old), boys, out of school, from the poorest households and those exposed to SHS. In Nigeria, a higher proportion of current users were aged 14–17 years, boys and those exposed to SHS. Full participant characteristics of current tobacco and/or nicotine use status are presented in online supplemental table 2.

#### Covariate balance and propensity score diagnostics

Prior to weighting, imbalances were observed for age, school attendance, household size and selected wealth categories. After weighting, all absolute SMDs were below 0.10 in the pooled and Kenya analyses, while household size in Nigeria remained slightly above the threshold (SMD=0.11). In the pooled analysis, the largest absolute weighted SMD was 0.07; across all pooled and country-specific analyses, the largest was 0.11. Weighted variance ratios ranged from 0.92 to 1.08 in the pooled analysis and from 0.90 to 1.10 across all analyses, remaining within the prespecified acceptable range of 0.80–1.25.^25,26^ Full balance diagnostics are presented in online supplemental table 3, and the Love plot is shown in online supplemental figure 2.

The propensity score distributions showed adequate overlap between adolescents exposed and unexposed to SHS in the pooled sample and in both country-specific analyses (online supplemental figure 3). The IPW treatment-weight distribution did not suggest extreme weighting. In the pooled analysis, the ATE treatment weights had a median of 1.87 (IQR: 1.42–2.14), with a 99th percentile of 4.54 and a maximum of 6.83. The ATT treatment weights had a median of 0.93 (IQR: 0.42–1.00), with a 99th percentile of 1.26 and a maximum of 2.30. These diagnostics support improved balance and adequate overlap after weighting, although they do not rule out residual unmeasured confounding.

#### IPW estimates of the association between SHS exposure and current tobacco and/or nicotine use

Table 2 presents the survey-weighted IPW risk-difference estimates for the association between SHS exposure and current tobacco and/or nicotine use. In the pooled analysis, adolescents exposed to SHS had a 2.65 percentage point higher probability of current tobacco and/or nicotine use compared with those not exposed, representing the estimated ATE. The ATT was of similar magnitude at a 2.42 percentage point increase in tobacco and/or nicotine use risk in the pooled analysis. Country-specific analyses showed smaller effects in Kenya (ATE: +1.32 pp; ATT: +1.16 pp) and larger effects in Nigeria (ATE: +3.94 pp; ATT: +4.21 pp). Both the ATE and ATT estimates were statistically significant and consistent in direction across all analyses (pooled, Kenya, Nigeria), indicating robustness to the choice of estimand.

**Table 2:** Survey-weighted IPW risk-difference estimates for the association between SHS exposure and current tobacco and/or nicotine use

Analysis group	ATE, percentage points (95% CI)	ATT, percentage points (95% CI)
Pooled	+2.65 (1.46 to 3.83)	+2.42 (1.35 to 3.49)
Kenya	+1.32 (0.19 to 2.45)	+1.16 (0.36 to 1.97)
Nigeria	+3.94 (1.95 to 5.93)	+4.21 (2.00 to 6.43)

ATE, average treatment effect; ATT, average treatment effect on the treated; IPW, inverse probability weighting; SHS, secondhand smoke.

#### Effect modification by country

Given the larger IPW estimates observed in Nigeria than Kenya, we formally tested for effect modification by country using an interaction term between SHS exposure and country in an IPW-weighted risk-difference model. There was evidence of effect modification by country on the additive risk-difference scale. Under the ATE specification, SHS exposure was associated with a 1.20 percentage point higher probability of current tobacco and/or nicotine use in Kenya, (95% CI 0.10 to 2.30), compared with 3.87 percentage points in Nigeria, (95% CI 1.93 to 5.81) (table 3). The Nigeria–Kenya difference in the SHS-associated risk difference was 2.67 percentage points (95% CI 0.44 to 4.91), interaction p value=0.019. Similar evidence of effect modification was observed under the ATT specification. The estimated ATT was 1.09 percentage points in Kenya, 95% CI 0.22 to 1.96 and 4.27 percentage points in Nigeria, 95% CI 2.11 to 6.44. The Nigeria–Kenya difference in ATT was 3.18 percentage points, 95% CI 0.85 to 5.52, interaction p value=0.008.

**Table 3:** Effect modification by country in the IPW-weighted RD model

Estimand	Kenya RD, pp (95% CI)	Nigeria RD, pp (95% CI)	Nigeria–Kenya difference, pp (95% CI)	Interaction p value
ATE	1.20 (0.10 to 2.30)	3.87 (1.93 to 5.81)	2.67 (0.44 to 4.91)	0.019
ATT	1.09 (0.22 to 1.96)	4.27 (2.11 to 6.44)	3.18 (0.85 to 5.52)	0.008

ATE, average treatment effect; ATT, average treatment effect on the treated; IPW, inverse probability weighting; RD, risk-difference.

#### Sensitivity analyses

To assess robustness, we examined the association between SHS exposure and current tobacco and/or nicotine use using multivariable survey-weighted logistic regression adjusting for the same covariates. SHS exposure remained associated with current tobacco and/or nicotine use. In the pooled model, adolescents exposed to SHS had more than double the odds of current tobacco and/or nicotine use (AOR=2.46 (1.75–3.45)). Country-specific analyses showed similar associations. In Nigeria, SHS exposure was linked to about three times higher odds (AOR=2.92 (2.01–4.25)), while in Kenya the odds of current use were 1.9 times higher (AOR=1.92; (1.20–3.07)). Adjusted predicted probabilities further supported this trend, showing a higher likelihood of tobacco use among exposed adolescents in both Kenya and Nigeria (online supplemental table 4).

To explore whether the binary SHS exposure definition masked differences by exposure context, we conducted an additional location-specific analysis using self-reported SHS exposure at home and in public places. Overall, 61.6% of adolescents reported no SHS exposure, 21.1% reported home-only exposure, 8.9% reported public-place-only exposure and 8.3% reported exposure both at home and in public places. After adjustment for demographic and socio-economic covariates, the predicted probability of current tobacco and/or nicotine use was highest among adolescents exposed both at home and in public places (7.8%) and among those exposed at home only (5.8%), compared with adolescents reporting no SHS exposure (2.1%). The adjusted risk difference was +3.7 percentage points for home-only exposure (95% CI 2.3 to 5.2) and +5.8 percentage points for exposure both at home and in public places (95% CI 1.8 to 9.7). Public-place-only exposure showed a lower adjusted predicted probability (risk difference: −1.0 percentage points; 95% CI −2.0 to −0.1), which should be interpreted cautiously because this analysis was exploratory and may be affected by residual confounding, exposure misclassification or differences in the characteristics of adolescents exposed only in public places. Pooled location-specific results are presented in online supplemental table 5.

Country-specific predicted probabilities suggested that the pattern was stronger in Nigeria. In Kenya, the adjusted predicted probability of current tobacco and/or nicotine use was 2.6% among adolescents with no SHS exposure, 3.9% among those exposed at home only, 0.5% among those exposed in public places only and 3.5% among those exposed both at home and in public places. In Nigeria, the corresponding predicted probabilities were 1.9%, 7.6%, 1.3% and 12.8%, respectively.

#### E-value analysis for unmeasured confounding

Because peer smoking and family or parental tobacco use were not available for the full analytical sample and could not be included in the primary propensity score model, we calculated E-values to assess the potential impact of unmeasured confounding. Using IPW-weighted risk ratios corresponding to the ATE analysis, the pooled risk ratio was 2.26 (95% CI 1.59 to 3.22), with an E-value of 3.95 for the point estimate and 2.55 for the confidence limit closest to the null. For Kenya, the corresponding risk ratio was 1.73 (95% CI 0.98 to 3.07), with an E-value of 2.86 for the point estimate and 1.00 for the confidence limit closest to the null. For Nigeria, the risk ratio was 2.69 (95% CI 1.86 to 3.88), with an E-value of 4.82 for the point estimate and 3.13 for the confidence limit closest to the null. These results indicate that, for the pooled and Nigeria estimates, an unmeasured confounder would need to be associated with both SHS exposure and current tobacco/nicotine use by at least these magnitudes, conditional on measured covariates, to fully explain away the observed association. For Kenya, the E-value for the confidence limit was 1.00 because the CI for the ATE-based risk ratio included the null. The complete E-value sensitivity-analysis results are presented in table 4.

**Table 4:** E-value sensitivity analysis for unmeasured confounding

Analysis group	Estimand	Risk unexposed, %	Risk exposed, %	IPW RR (95% CI)	E-value	E-value for CI limit
Pooled	ATE	2.10	4.75	2.26 (1.59 to 3.22)	3.95	2.55
Kenya	ATE	1.81	3.13	1.73 (0.98 to 3.07)	2.86	1.00
Nigeria	ATE	2.33	6.27	2.69 (1.86 to 3.88)	4.82	3.13

ATE, average treatment effect; IPW, inverse probability weighting; RR, risk ratio.

## Discussion

This study found that self-reported SHS exposure was associated with a higher probability of current tobacco and/or nicotine use among adolescents in Kenya and Nigeria. Using survey-weighted IPW, we found a significant increase in current use among exposed adolescents in pooled and country-specific analyses, with larger estimates observed in Nigeria. These findings were consistent across ATE and ATT estimates and were supported by multivariable logistic regression, which showed more than double the odds of current use among exposed adolescents in the pooled sample (AOR 2.5), Kenya (1.9) and Nigeria (2.9). Together, these findings point to SHS exposure as an important modifiable risk factor that can be targeted in tobacco control interventions. The ATT estimates are particularly relevant for policy because they quantify the excess probability of current tobacco and/or nicotine use among adolescents already exposed to SHS, a group that may benefit most immediately from strengthened smoke-free protections.

Our findings align with previous research showing that SHS exposure increases tobacco use susceptibility and uptake among adolescents. A systematic review on secondhand tobacco smoke exposure and smoking behaviours reported consistent associations with smoking initiation, dependence and susceptibility.^27^ Similarly, evidence from a prospective cohort study in China found that SHS exposure doubled the risk of adolescent smoking uptake.^5^ Analyses of Global Youth Tobacco Survey data also show that SHS exposure at home increased smoking susceptibility,^10,19^ and a study in Lagos showed that adolescents exposed to indoor SHS had nearly four times the odds of intending to smoke^20^ while Hong *et al* also identified SHS exposure as a key driver of adolescent tobacco use in low- and middle-income countries.^18^ While these studies were largely associative, our study extends this body of evidence by applying a causal-inference framework, assessing covariate balance, examining propensity score overlap and conducting sensitivity analyses for unmeasured confounding in a SSA adolescent population, where such evidence remains limited.

Although SHS exposure was more common in Kenya (48.7%) than in Nigeria (29.2%), the estimated association was about three times higher in Nigeria. The formal interaction analysis supported effect modification by country, with the SHS-associated risk difference significantly larger in Nigeria than Kenya. Several factors may explain this pattern. First, the location-specific analysis suggested that exposure at home and combined exposure at home and in public places were associated with the highest predicted probabilities of current tobacco and/or nicotine use, and this pattern was particularly marked in Nigeria. This may indicate that more proximal or repeated exposures, especially in household settings carry stronger behavioural influence than brief or occasional encounters in public settings.^5^ Second, SHS exposure may operate partly through social normalisation of tobacco use, reduced perceived harm, increased access to tobacco products or modelling of smoking behaviour by adults or peers. In Kenya, the higher exposure prevalence may reflect shorter public-space encounters, which offer less sustained behavioural influence.^5,28^

However, the stronger association observed in Nigeria should be interpreted cautiously. Peer smoking and family or parental tobacco use were not available for the full analytical sample and could not be included in the primary propensity score model. These factors may influence both SHS exposure and adolescent tobacco or nicotine use and may partly explain the larger Nigeria estimate. The E-value analysis suggested that the pooled and Nigeria estimates would require moderately strong unmeasured confounding to be fully explained away, but it does not eliminate the possibility of residual confounding. For Kenya, the E-value for the confidence limit closest to the null was 1.00 because the ATE-based risk-ratio CI included the null, indicating that the Kenya estimate was less robust to unmeasured confounding than the pooled and Nigeria estimates.

Policy enforcement differences may also contribute to the country-level variation observed in our results. Both countries are signatories to the WHO FCTC, but enforcement varies. Kenya has long-standing national smoke-free laws, yet exposure remains widespread, pointing to enforcement gaps.^29^ Nigeria’s smoke-free regulations are more recent and unevenly enforced across states. These policy and contextual differences may shape both exposure patterns and behavioural outcomes, contributing to the country-level variation observed in our results.

### Strengths and limitations of the study

This study addresses critical evidence gaps in adolescent tobacco use research in SSA by applying a causal inference framework to nationally representative survey data. The use of survey-weighted IPW allowed us to estimate risk differences after improving balance across measured demographic, socio-economic and household characteristics. The consistency of findings across ATE and ATT estimates, multivariable logistic regression, location-specific analyses and E-value sensitivity analyses strengthens the robustness of the overall interpretation.

However, the study’s cross-sectional design limits causal claims about temporality. It is possible that adolescents who already use tobacco and/or nicotine products are more likely to spend time with peers or adults who smoke, thereby increasing their reported SHS exposure. This reciprocal relationship could lead to upward bias in the estimated association. In addition, self-reported SHS exposure and tobacco and/or nicotine use may be affected by recall or social desirability bias. Although adjustment was made for a comprehensive set of observed confounders, unmeasured confounding cannot be ruled out. The binary SHS exposure measure did not capture exposure duration, intensity or setting, potentially masking dose response relationships or context-specific effects. Our location-specific analysis partly addressed this concern and suggested stronger associations for home-only and combined home and public exposure. However, this analysis was exploratory, and the unexpected lower predicted probability among adolescents exposed only in public places should not be interpreted as protective, as it may reflect residual confounding, exposure misclassification, lower exposure intensity or differences in the characteristics of adolescents exposed only in public places. Future research should adopt longitudinal designs and capture detailed exposure contexts.

### Policy implications

These findings underscore the need for stronger, context-specific tobacco control measures in Kenya and Nigeria. Although both countries have adopted WHO FCTC provisions, the persistence of adolescent SHS exposure suggests that current smoke-free laws do not adequately protect young people in youth-centric environments such as schools, recreational venues, homes and public transport. The stronger association observed in Nigeria suggests that prevention strategies may need to pay particular attention to high-risk household and social environments, while the higher prevalence of SHS exposure in Kenya highlights the need for broad enforcement of smoke-free protections.

Given the observed association between SHS exposure and adolescent tobacco and/or nicotine use, interventions must go beyond policy development to prioritise enforcement and behaviour change. Campaigns targeting parents and caregivers should raise awareness of the behavioural risk of SHS, and support smoke-free homes including cessation services could serve as effective tools. Because current tobacco and/or nicotine use was more common among out-of-school adolescents, prevention should not rely only on school-based programmes but should also include community-based outreach, youth centres, recreational spaces and household-level interventions. By identifying SHS exposure as a modifiable risk marker for adolescent tobacco and/or nicotine use, this study highlights the urgency of implementing and enforcing comprehensive smoke-free environments. Protecting adolescents from involuntary SHS exposure is not only an immediate public health priority but also a strategic long-term investment in reducing tobacco-related harm across the life course in SSA.

## Supplementary material

10.1136/bmjph-2025-004773 online supplemental file 1

## Data Availability

Data are available in a public, open access repository.

## References

[1] World Health Organization Tobacco and nicotine 2026. https://www.who.int/news-room/fact-sheets/detail/tobacco.

[2] Ghebreyesus TA, Bloomberg MR, Krech R (2023). WHO report on the global tobacco epidemic.

[3] Lee S, Son Y, Hong S (2024). Global trends of secondhand smoke exposure among young adolescents from 27 countries, 2003–2021: findings from the World Health Organization database. Sci Rep.

[4] Altwicker-Hámori S, Ackermann KA, Furchheim P (2024). Risk factors for smoking in adolescence: evidence from a cross-sectional survey in Switzerland. BMC Public Health.

[5] Yang X, Yan Z, Xu G (2021). How secondhand smoke exposure affects tobacco use and smoking susceptibility of adolescents: Sex and school differences. Tob Induc Dis.

[6] Mazi AA (2025). Second-hand smoke exposure among school children during COVID-19 in Jeddah. *J Taibah Univ Med Sci*.

[7] Huai B, Chang KCM, Filippidis FT (2024). Inequalities in exposure to second-hand smoke among adolescent boys and girls in 122 countries: Evidence from the Global Youth Tobacco Survey. Prev Med.

[8] Ma C, Huang Y, Li S (2023). Trends in Exposure to Secondhand Smoke Among Adolescents in China From 2013-2014 to 2019: Two Repeated National Cross-sectional Surveys. JMIR Public Health Surveill.

[9] Ma C, Heiland EG, Li Z (2021). Global trends in the prevalence of secondhand smoke exposure among adolescents aged 12-16 years from 1999 to 2018: an analysis of repeated cross-sectional surveys. Lancet Glob Health.

[10] James PB, Bah AJ, Kabba JA (2022). Prevalence and correlates of current tobacco use and non-user susceptibility to using tobacco products among school-going adolescents in 22 African countries: a secondary analysis of the 2013-2018 global youth tobacco surveys. Arch Public Health.

[11] Mbulo L, Palipudi K, Smith T (2023). Secondhand Smoke Exposure Inside the Home Among Adults in Eight Countries in Sub-Saharan Africa: Global Adult Tobacco Survey, 2012-2018. Nicotine Tob Res.

[12] Omaduvie U, Adisa A (2015). Exposure to Secondhand Smoke in the Home and Public areas among Adolescents in Abuja, Nigeria: Tobacco control implications. Tob Prev Cessation.

[13] Ministry of Health (MoH) (2013). Global youth tobacco survey - fact sheet.

[14] Bandura A (1977). Social Learning Theory. Illustrate.

[15] Heo J, Oh J, Subramanian SV (2014). Household and school-level influences on smoking behavior among Korean adolescents: a multilevel analysis. PLoS One.

[16] Orton S, Jones LL, Cooper S (2014). Predictors of children’s secondhand smoke exposure at home: a systematic review and narrative synthesis of the evidence. PLoS One.

[17] Do EK, Bradley KC, Fugate-Laus K (2021). An examination of social and environmental determinants of secondhand smoke exposure among non-smoking adolescents. Tob Prev Cessat.

[18] Hong S, Son Y, Lee H (2024). Global association of secondhand smoke exposure locations and smoking behaviour among adolescents in 99 countries. Acta Paediatr.

[19] Kyung AL, Krishna MP, Lorna ME (2016). Secondhand smoke exposure and susceptibility to initiating cigarette smoking among never-smoking students in selected African countries: Findings from the Global Youth Tobacco Survey. Prev Med (Baltim).

[20] Oyapero A, Erinoso O, Olatosi OO (2023). Adolescents’ exposure to secondhand smoke and its association with susceptibility to smoking and mental health in Lagos, Nigeria. Pan Afr Med J.

[21] Itanyi IU, Onwasigwe CN, Ossip D (2020). Predictors of current tobacco smoking by adolescents in Nigeria: Interaction between school location and socioeconomic status. Tob Induc Dis.

[22] Sunday S, Clancy L, Hanafin J (2023). The associations of parental smoking, quitting and habitus with teenager e-cigarette, smoking, alcohol and other drug use in GUI Cohort ’98. Sci Rep.

[23] Kisia L, Mohamed SF, Kyule G (2024). Use of tobacco and nicotine products among adolescents in sub-saharan africa: protocol for a population-based multi-country household survey. Public and Global Health.

[24] Mohamed SF (2024). Primary Research for the Data on Youth and Tobacco in Africa (DaYTA) program, DaYTA: Kenya, Nigeria and Democratic Republic of the Congo. https://microdataportal.aphrc.org/index.php/catalog/215.

[25] Austin PC, Stuart EA (2015). Moving towards best practice when using inverse probability of treatment weighting (IPTW) using the propensity score to estimate causal treatment effects in observational studies. Stat Med.

[26] Rubin DB (2006). Using propensity scores to help design observational studies: Application to the tobacco litigation. Matched Sampling for Causal Effects.

[27] Okoli CTC, Kodet J (2015). A systematic review of secondhand tobacco smoke exposure and smoking behaviors: Smoking status, susceptibility, initiation, dependence, and cessation. Addict Behav.

[28] Veeranki SP, Mamudu HM, Zheng S (2015). Secondhand smoke exposure among never-smoking youth in 168 countries. J Adolesc Health.

[29] Kenya law (2012). Tobacco control act.

