# Supplementary material for: Secondhand smoke exposure and current tobacco and/or nicotine use among adolescents in Kenya and Nigeria: a survey-weighted inverse probability weighting analysis
Source: BMJ Public Health. 2026 Oct 13;4(4):e004773. doi: 10.1136/bmjph-2025-004773 (PMC13663890; doi:10.1136/bmjph-2025-004773)

Supplemental Materials

Manuscript ID: bmjph-2025-004773.R1
Article title: Second-hand smoke exposure and current tobacco and/or nicotine use among adolescents in Kenya and Nigeria: A survey-weighted inverse probability weighting analysis
Corresponding author: Grace Kyule
Contents: Supplementary Tables 1–5 and Supplementary Figures 1–3

Supplementary Tables

Supplementary Table 1: Full participant characteristics by second-hand smoke (SHS) exposure status, pooled and by country.

	Pooled N (13,937)			Kenya N (6,435)			Nigeria N (7,502)		
**Characteristic**	**Not exposed**	**Exposed**	**p-value**	**Not exposed**	**Exposed**	**p-value**	**Not exposed**	**Exposed**	**p-value**
**SHS exposure, n (%)**	8,606 (61.8)	5,331 (38.2)		3,245 (51.3)	3,190 (48.7)		5,361 (70.8)	2,141 (29.2)	
**Age group, n (%)**									
10-13 years	5,059 (63.8)	2,888 (36.2)	0.006	1,769 (52.2)	1,712 (47.8)	0.37	3,290 (73.3)	1,176 (26.7)	0.008
14-17 years	3,547 (59.4)	2,443 (40.6)		1,476 (50.3)	1,478 (49.7)		2,071 (67.6)	965 (32.4)	
**Sex, n (%)**									
Girls	4,351 (62.5)	2,561 (37.5)	0.245	1,762 (54.0)	1,577 (46.0)	0.001	2,589 (70.4)	984 (29.6)	0.636
Boys	4,255 (61.1)	2,770 (38.9)		1,483 (48.5)	1,613 (51.5)		2,772 (71.1)	1,157 (28.9)	
**Residence, n (%)**									
Rural	5,234 (60.2)	3,420 (39.9)	0.01	2,072 (51.0)	2,099 (49.0)	0.544	3,162 (71.9)	1,321 (28.1)	0.312
Urban	3,372 (64.5)	1,911 (35.5)		1,173 (52.2)	1,091 (47.8)		2,199 (69.6)	820 (30.4)	
**School status, n (%)**									
Out of school	1,223 (73.0)	424 (27.0)	<0.001	222 (64.4)	143 (35.6)	<0.001	1,001 (76.4)	281 (23.6)	0.02
In school	7,364 (59.9)	4,897 (40.1)		3,022 (50.0)	3,045 (50.0)		4,342 (69.5)	1,852 (30.5)	
**Work status, n (%)**									
Not employed	8,011 (62.4)	4,827 (37.6)	0.005	3,107 (52.2)	2,964 (47.8)	<0.001	4,904 (71.6)	1,863 (28.4)	0.02
Employed	564 (55.3)	492 (44.7)		138 (38.5)	225 (61.5)		426 (63.8)	267 (36.2)	
**Household head education, n (%)**									
Primary and below	3,726 (61.9)	2,353 (38.1)	0.536	1,622 (52.9)	1,586 (47.1)	0.171	2,104 (72.6)	767 (27.4)	0.213

	Pooled N (13,937)			Kenya N (6,435)			Nigeria N (7,502)		
**Characteristic**	**Not exposed**	**Exposed**	**p-value**	**Not exposed**	**Exposed**	**p-value**	**Not exposed**	**Exposed**	**p-value**
Secondary +	3,866 (62.9)	2,312 (37.1)		1,182 (50.0)	1,185 (50.0)		2,684 (69.7)	1,127 (30.3)	
**Wealth quintile, n (%)**									
Poorest	1,841 (66.9)	949 (33.1)	<0.001	745 (56.9)	648 (43.2)	0.006	1,096 (75.5)	301 (24.5)	0.03
Poor	1,631 (59.2)	1,155 (40.8)		781 (49.2)	794 (50.8)		850 (70.3)	361 (29.7)	
Middle	1,618 (56.8)	1,169 (43.3)		690 (48.0)	715 (52.0)		928 (66.6)	454 (33.4)	
Rich	1,667 (60.1)	1,121 (39.9)		608 (49.7)	641 (52.3)		1,059 (69.3)	480 (30.7)	
Richest	1,848 (64.8)	937 (35.2)		421 (52.3)	392 (47.7)		1,427 (70.0)	545 (30.0)	
**Household size Median (IQR)**	4 (3-5)	4 (3-5)		4 (3-5)	4 (3-5)		3 (3-4)	3 (3-5)	
**Current tobacco and/or nicotine use, n (%)**									
No	8,445 (62.3)	5,076 (37.7)	<0.001	3,209 (51.4)	3,111 (48.6)	0.463	5,236 (71.7)	1,965 (28.3)	<0.001
Yes	161 (46.5)	255 (53.5)		36 (45.8)	79 (54.3)		125 (47.0)	176 (53.0)	

Notes: values are unweighted frequencies (n) and survey weighted percentages (%) unless otherwise stated. Percentages are row percentages showing the distribution of SHS exposure within each participant characteristic. P-values are design-adjusted p-values comparing adolescents exposed and not exposed to SHS within the pooled sample and within each country; p-values <0.001 are reported as <0.001.

Supplementary Table 2: Participant characteristics by current use of tobacco and/or nicotine.

Characteristic	Pooled N (13,937)			Kenya N (6,435)			Nigeria N (7,502)		
	No current use	Current use	p-value	No current use	Current use	p-value	No current use	Current use	p-value
**Age group, n (%)**									
10-13 years	7,819 (54.6)	128 (35.6)	<0.001	3,444 (53.0)	37 (37.3)	0.014	4,375 (55.9)	91 (34.6)	<0.001
14-17 years	5,702 (45.4)	288 (64.6)		2,876 (47.0)	28 (62.7)		2,826 (44.1)	210 (65.4)	
**Sex, n (%)**									
Girls	6,798 (50.1)	114 (27.9)	<0.001	3,301 (51.7)	38 (36.7)	0.002	3,497 (48.7)	76 (22.7)	<0.001
Boys	6,723 (49.9)	302 (72.1)		3,019 (48.3)	77 (63.3)		3,704 (51.3)	225 (77.3)	
**Residence, n (%)**									
Rural	8,381 (62.9)	273 (64.3)	0.789	4,088 (76.4)	83 (84.2)	0.209	4,293 (51.2)	190 (52.5)	0.819
Urban	5,140 (37.1)	143 (35.7)		2,232 (23.6)	32 (15.8)		2,908 (48.8)	111 (47.5)	
**School status, n (%)**									
Out of school	1,535 (13.2)	112 (38.9)	<0.001	314 (7.6)	51 (63.2)	<0.001	1,221 (19.3)	61 (24.5)	0.167
In school	11,957 (86.1)	304 (61.1)		6,003 (92.4)	64 (36.8)		5,954 (80.7)	240 (75.5)	
**Work status, n (%)**									
Not employed	12,517 (91.3)	321 (78.1)	<0.001	5,975 (93.5)	96 (87.5)	0.059	6,542 (89.3)	225 (72.4)	<0.001
Employed	964 (8.7)	92 (21.9)		344 (6.5)	19 (12.5)		620 (10.7)	73 (27.6)	
**Household head education, n (%)**									
Primary and below	5,906 (56.8)	173 (55.9)	0.877	3,138 (66.8)	70 (86.0)	0.004	2,768 (48.3)	103 (39.2)	0.069
Secondary +	5,983 (43.2)	195 (44.1)		2,342 (33.2)	25 (14.0)		3,641 (51.7)	170 (60.8)	
**Wealth quintile, n (%)**									
Poorest	2,677 (25.4)	113 (38.7)	0.029	1,336 (24.5)	57 (68.1)	<0.001	1,341 (26.2)	56 (21.3)	0.468
Poor	2,716 (22.0)	70 (17.2)		1,557 (25.2)	18 (10.3)		1,159 (19.3)	52 (21.3)	
Middle	2,701 (20.0)	86 (16.9)		1,388 (23.1)	17 (11.1)		1,313 (17.3)	69 (20.3)	
Rich	2,721 (16.6)	67 (10.7)		1,233 (17.0)	16 (8.1)		1,488 (16.4)	51 (12.3)	
Richest	2,705 (16.0)	80 (16.5)		806 (10.3)	7 (2.4)		1,899 (20.9)	73 (24.8)	
**Household size median (IQR)**	4 (3-5)	4 (3-5)		4 (3-5)	4 (3-5)		3 (3-5)	4 (3-5)	
**Mean (sd)**	3.9 (1.6)	3.9 (1.6)		4.2 (1.7)	4.1 (1.9)		3.6 (1.5)	3.8 (1.5)	
**Current tobacco or nicotine use, n (%)**	13,521 (96.9)	416 (3.1)		6,320 (97.5)	115 (2.5)		7,201 (96.4)	301 (3.7)	
**SHS exposure, n (%)**									

	Pooled N (13,937)			Kenya N (6,435)			Nigeria N (7,502)		
**Characteristic**	**No current use**	**Current use**	**p-value**	**No current use**	**Current use**	**p-value**	**No current use**	**Current use**	**p-value**
Not exposed	8,445 (62.3)	161 (46.5)	<0.001	3,209 (51.4)	36 (45.8)	0.463	5,236 (71.7)	125 (47.0)	<0.001
Exposed	5,076 (37.7)	255 (53.5)		3,111 (48.6)	79 (54.3)		1,965 (28.3)	176 (53.0)	

Notes: values are unweighted frequencies (n) and survey-weighted percentages (%) unless otherwise stated. Percentages are column percentages showing the distribution of participant characteristics among adolescents with and without current tobacco and/or nicotine use. Current tobacco and/or nicotine use was defined as self-reported use of any tobacco and/or nicotine product in the past 30 days. P-values are design-adjusted p-values comparing adolescents with and without current tobacco and/or nicotine use within the pooled sample and within each country. P-values <0.001 are reported as <0.001. Percentages may not sum to 100 because of rounding.

Supplementary Table 3: Covariate balance before and after weighting (IPW) pooled and stratified by country

Covariate	Metric	Pooled	Kenya	Nigeria
**Age ≥14 yrs**	SMD Raw	0.11	0.03	0.14
	SMD Weighted	-0.004	-0.01	-0.01
	VR Raw	1.03	1.01	1.05
	VR Weighted	1.00	0.99	0.99
**Male (Sex)**	SMD Raw	0.03	0.07	0.03
	SMD Weighted	0.03	-0.00	0.06
	VR Raw	1.00	1.00	0.99
	VR Weighted	1.00	1.00	0.99
**Urban residence**	SMD Raw	-0.05	-0.02	-0.05
	SMD Weighted	-0.06	-0.02	-0.09
	VR Raw	0.97	0.99	0.98
	VR Weighted	0.97	0.99	0.96
**In school**	SMD Raw	0.22	0.12	0.17
	SMD Weighted	0.03	-0.01	0.06
	VR Raw	0.57	0.62	0.72
	VR Weighted	0.92	1.05	0.90
**Adolescent work**	SMD Raw	0.10	0.12	0.15
	SMD Weighted	0.02	0.01	0.04
	VR Raw	1.37	1.62	1.49
	VR Weighted	1.08	1.03	1.10
**Head education**	SMD Raw	-0.03	0.01	0.07
	SMD Weighted	-0.02	-0.04	0.01
	VR Raw	1.00	1.00	0.98
	VR Weighted	1.00	0.99	1.00
**HH size**	SMD Raw	0.19	0.07	0.16
	SMD Weighted	0.07	0.01	0.11
	VR Raw	1.09	0.95	1.22
	VR Weighted	0.99	0.92	1.10
**Wealth: Poor**	SMD Raw	0.06	0.01	0.02
	SMD Weighted	0.01	-0.03	0.04
	VR Raw	1.09	1.01	1.03
	VR Weighted	1.02	0.97	1.08
**Wealth: Middle**	SMD Raw	0.10	0.04	0.12
	SMD Weighted	0.02	-0.02	0.04
	VR Raw	1.15	1.06	1.21
	VR Weighted	1.02	0.98	1.06
**Wealth: Rich**	SMD Raw	0.04	0.04	0.06
	SMD Weighted	0.02	-0.00	0.04
	VR Raw	1.07	1.06	1.10
	VR Weighted	1.03	0.99	1.06
**Wealth: Richest**	SMD Raw	-0.09	0.01	-0.02
	SMD Weighted	-0.05	0.00	-0.07
	VR Raw	0.87	1.02	0.98
	VR Weighted	0.93	1.01	0.92
**Country: Nigeria**	SMD Raw	-0.46		
	SMD Weighted	-0.04		
	VR Raw	1.03		
	VR Weighted	1.01		

Notes: SMD, standardised mean difference; VR, variance ratio; IPW, inverse probability weighting. Raw and weighted values represent estimates before and after weighting, respectively. Adequate balance was defined as |SMD|<0.10 and VR 0.80–1.25. Country was included only in the pooled propensity-score model.

Supplementary Table 4: Adjusted predicted probability of current tobacco and/or nicotine use by SHS exposure and country

Country	SHS exposure	Predicted probability (%)	95% CI
**Kenya**	Not exposed	2.64	(0.88, 4.40)
	Exposed	3.29	(1.76, 4.82)
	Risk difference	+0.65	
**Nigeria**	Not exposed	1.90	(1.34, 2.45)
	Exposed	6.17	(4.41, 7.92)
	Risk difference	+4.27	

Notes: Predicted probabilities were estimated from the survey-weighted multivariable logistic regression models. Risk differences represent the predicted probability among SHS-exposed adolescents minus that among unexposed adolescents. CI, confidence interval.

Supplementary Table 5: Adjusted predicted probability of current tobacco and/or nicotine use by SHS exposure location

**SHS exposure location**	**Adjusted predicted probability, % (95% CI)**	**Risk difference vs no SHS, pp (95% CI)**
No SHS exposure	2.1 (1.3, 2.9)	Reference
Home only	5.8 (4.2, 7.4)	+3.7 (2.3, 5.2)
Public places only	1.0 (0.3, 1.7)	-1.0 (-2.0, -0.1)
Home and public places	7.8 (3.9, 11.8)	+5.8 (1.8, 9.7)

Supplementary Figures

Supplementary Figure 1: Directed acyclic graph for the association between second-hand smoke exposure and current tobacco and/or nicotine use among adolescents.

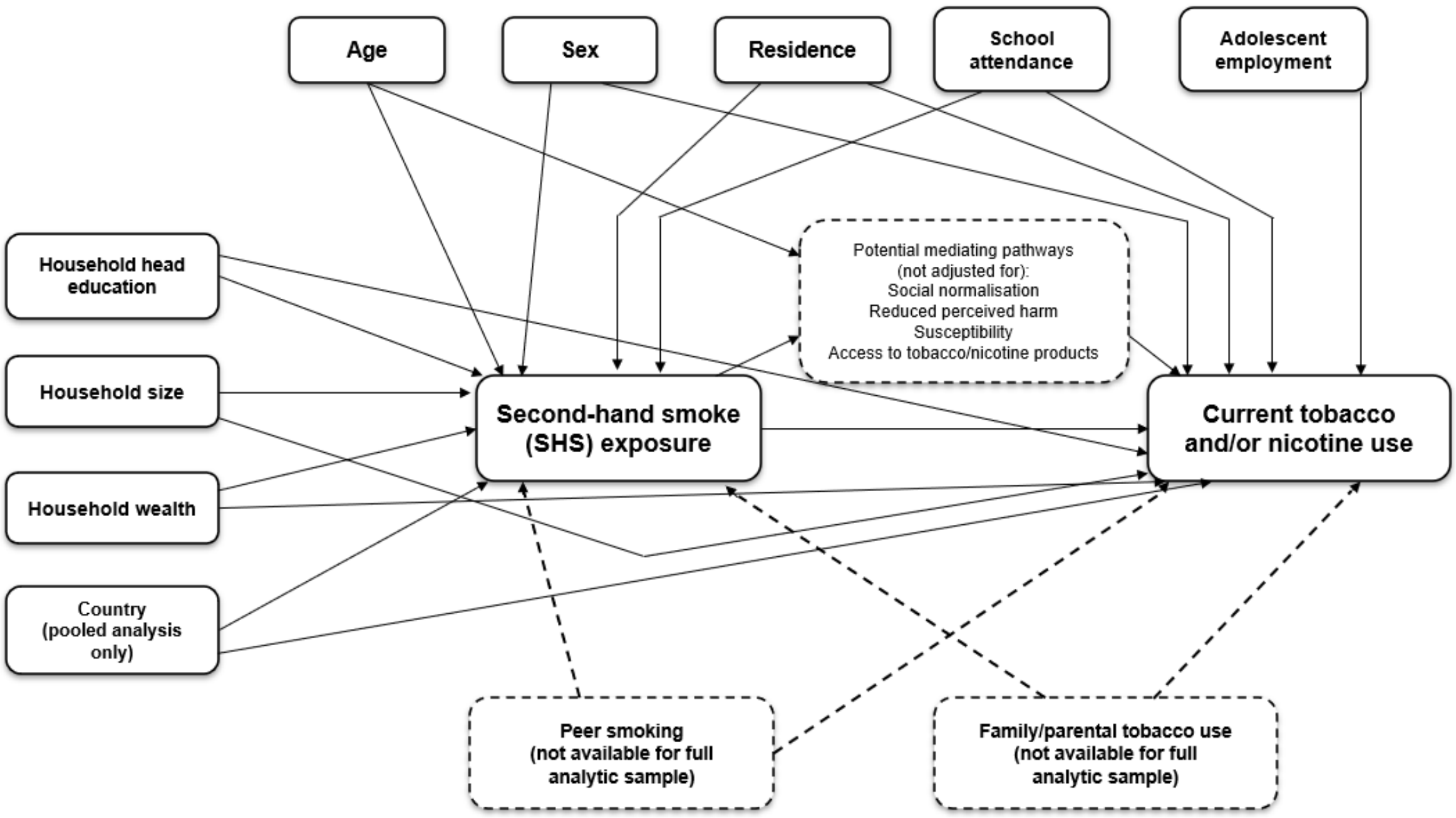

Note: solid boxes and arrows represent measured variables included in the primary propensity score model. Dashed boxes and arrows represent peer smoking and family/parental tobacco use, which were considered important potential confounders but were not available for the full analytic sample. The dashed mediating pathway box represents possible mechanisms that were not adjusted for in the primary analysis. Country was included only in the pooled analysis.

Supplementary Figure 2: Love plot showing covariate balance before and after inverse probability weighting

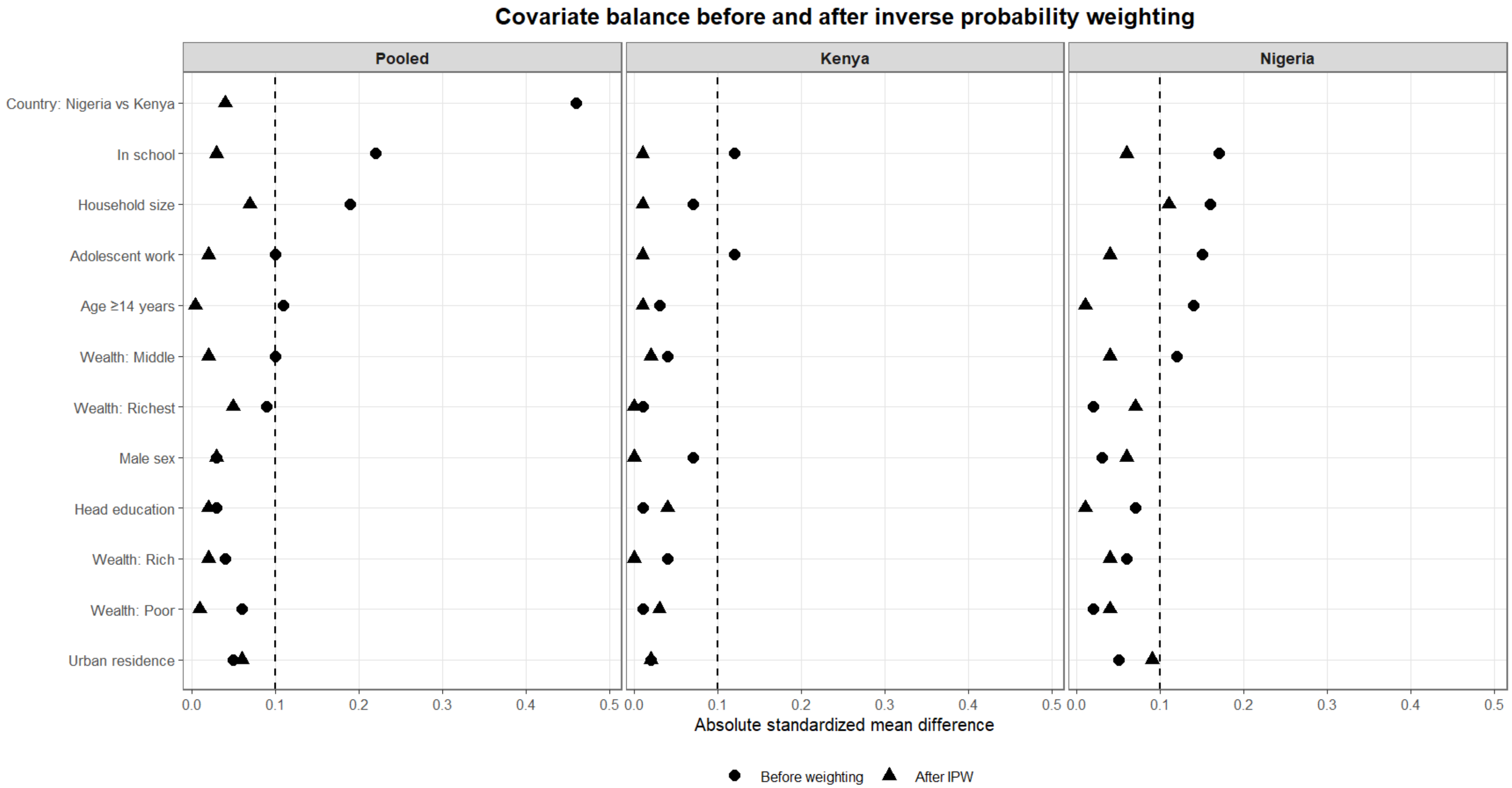

Dashed line indicates the 0.10 threshold for acceptable covariate balance. Country is included only in the pooled model.

Supplementary Figure 3: Propensity score overlap by second-hand smoke exposure status

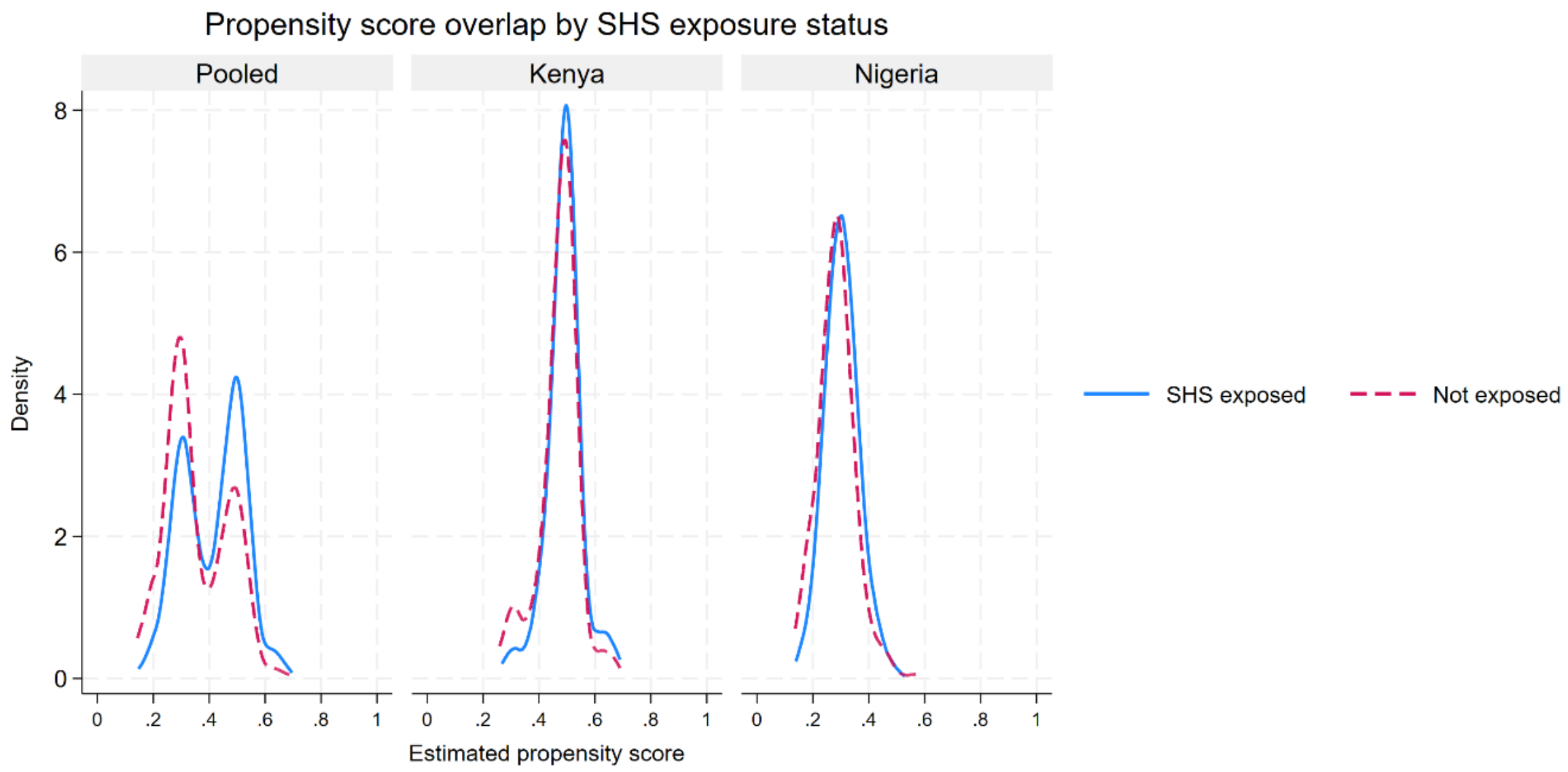

Supplement: Supplementary file 1 [file bmjph-4-4-s001.pdf]
